# Novel Therapy for Atherosclerosis Using Recombinant Immunotoxin Against Folate Receptor β–Expressing Macrophages

**DOI:** 10.1161/JAHA.112.003079

**Published:** 2012-08-24

**Authors:** Yuko Furusho, Masaaki Miyata, Takami Matsuyama, Taku Nagai, Hua Li, Yuichi Akasaki, Narisato Hamada, Takahiro Miyauchi, Yoshiyuki Ikeda, Takahiro Shirasawa, Kanako Ide, Chuwa Tei

**Affiliations:** Department of Cardiovascular, Respiratory, and Metabolic Medicine, Kagoshima University, Kagoshima, Japan (Y.F., M.M., Y.A., N.H., T. Miyauchi, Y.I., T.S., K.I., C.T.); Department of Immunology, Graduate School of Medicine, Kagoshima University, Kagoshima, Japan (T. Matsuyama, T.N., H.L.)

**Keywords:** antibodies, atherosclerosis, inflammation, plaque, receptors

## Abstract

**Background:**

Folate receptor β (FRβ) is induced during macrophage activation. A recombinant immunotoxin consisting of the truncated *Pseudomonas* exotoxin A (PE38) conjugated to an anti-FRβ antibody (anti–FRβ-PE38) has been reported to kill activated macrophages in inflammatory diseases. To elucidate the effect of an immunotoxin targeting FRβ on atherosclerosis, we determined the presence of FRβ-expressing macrophages in atherosclerotic lesions and administered the FRβ immunotoxin in apolipoprotein E–deficient mice.

**Methods and Results:**

The FRβ-expressing macrophages were observed in atherosclerotic lesions of apolipoprotein E–deficient mice. At 15 or 35 weeks of age, the apolipoprotein E–deficient mice were divided into 3 groups and were intravenously administered 0.1 mg/kg of anti–FRβ-PE38 (immunotoxin group), 0.1 mg/kg of PE38 (toxin group), or 0.1 mL of saline (control group) every 3 days, for a total of 5 times for each age group. The mice were analyzed at 21 or 41 weeks of age. Treatment with the immunotoxin resulted in 31% and 22% reductions in atherosclerotic lesions of the 21- and 41-week-old mice, respectively (*P*<0.05). Administration of immunotoxin reduced the numbers of FRβ- and tumor necrosis factor-α–expressing macrophages, reduced cell proliferation, and increased the number of apoptotic cells (*P*<0.05). Real-time polymerase chain reaction demonstrated that the expression of FRβ and tumor necrosis factor-α mRNA was significantly decreased in the immunotoxin group (*P*<0.05).

**Conclusions:**

These results suggest that FRβ-expressing macrophages exist in the atherosclerotic lesions of apolipoprotein E–deficient mice and that FRβ immunotoxin administration reduces the progression of atherosclerotic lesions in younger and older individuals. The recombinant FRβ immunotoxin targeting activated macrophages could provide a novel therapeutic tool for atherosclerosis. **(*J Am Heart Assoc*. 2012;1:e003079 doi: 10.1161/JAHA.112.003079.)**

## Introduction

Atherosclerosis is a chronic inflammatory disease in which activated macrophages play an important role by producing cytokines, chemokines, proteolytic enzymes, and growth factors.^1–2^ Induction of macrophage apoptosis during the early stage of atherosclerosis reduces lesion progression.^3^ In contrast, induction of macrophage apoptosis in the advanced stage contributes to the lipid core of an atheroma and leads to the progression of atherosclerotic lesions.^4–5^ Thus, the antiatherogenic effect of inducing macrophage apoptosis is controversial because the consequences of macrophage apoptosis differ according to the stage of the lesion.^6^

A folate receptor (FR) is a glycoprotein that is anchored to various cells by a glycosylphosphatidylinositol linkage.^7^ The FR exhibits a high affinity for folic acid and mediates its transmembrane transport into the cytoplasm for biosynthesis of purine and pyrimidine.^8^ The FR has at least 3 isoforms (α, β, γ).^9–10^ Previously, we showed that FRβ is induced during macrophage activation and that FRβ expression is a characteristic of activated macrophages.^11^ Furthermore, we demonstrated that a recombinant immunotoxin consisting of the truncated *Pseudomonas* exotoxin A (PE38) conjugated to a disulfide-stabilized fragment of the variable region of an anti-FRβ antibody (dsFv anti–FRβ-PE38) killed activated macrophages in rheumatoid arthritis and experimental models of pulmonary fibrosis.^12–13^

It recently has been reported that because the FR is a marker of macrophage activation, FR-expressing macrophages represent a specific target for folate-linked drugs, given that they would not be taken up by nonactivated macrophages.^14^ It also has been reported that macrophages taking up folate accumulate in the atherosclerotic lesions of apolipoprotein E (apoE)–deficient mice.^15^ We therefore hypothesized that depletion of FRβ-expressing macrophages via the dsFv anti–FRβ-PE38 immunotoxin could be beneficial in treating atherosclerosis by specifically targeting activated macrophages.

The purpose of the present study was to determine the presence of FRβ-expressing macrophages in atherosclerotic lesions and to investigate the effect of the recombinant anti–FRβ-PE38 immunotoxin administered at an early or late stage of atherosclerosis in a murine model of experimental atherosclerosis.

## Methods

### Experimental Animals

ApoE-deficient mice on a C57BL/6J background were kindly provided by Dr Jan L. Breslow (Rockefeller University, New York, NY). The mice were maintained in a temperature-controlled facility and fed a regular mouse chow diet. All animal procedures were performed in accordance with the Ethical Guideline for Animal Experiments, Kagoshima University, and were approved by the Kagoshima University Committee. The study conformed to the Guide for the Care and Use of Laboratory Animals published by the United States National Institutes of Health (NIH Publication No. 85-23, revised 1996).

### Production of Anti-Mouse FRβ Monoclonal Antibodies

Rat anti-mouse monoclonal antibodies (mAbs) against FRβ were produced in our laboratory as previously described.^12,16^ Briefly, mouse FRβ complementary DNA (cDNA) was prepared from a reverse transcription–polymerase chain reaction (PCR) product derived from BALB/c mouse liver. Primer sequences used were as follows: 5′-TCTAGAAAGACATGGCCTGGAAACAG-3′ (upstream) and 5′-CCCAACATGGATCAGGAACT-3′ (downstream). Wistar-Kyoto rats (Charles River Laboratories, Yokohama, Japan) were immunized with RBL2H3 (rat mastocytoma) cells transfected with the mouse FRβ gene. Lymphocytes from the iliac lymph nodes were fused with NS-1 myeloma cells. Hybridomas were screened for reactivity with FRβ-transfected RBL2H3 cells by flow-cytometric and Western blot analyses.

### Production of a Recombinant Immunotoxin Against Mouse FRβ

The recombinant dsFv anti–FRβ-PE38 immunotoxin, which consists of the immunoglobulin (Ig) heavy-chain Fv portion of the anti-mouse FRβ mAb (IgV_L_) with PE38 (V_H_-PE38) and the Ig light-chain Fv portion of anti-mouse FRβ mAbs, was prepared as previously described.^16–17^ In brief, inclusion bodies from bacteria transfected with expression plasmids encoding the IgV_H_-PE38 and IgV_L_ genes were solubilized separately and then combined. Properly folded dsFv anti–FRβ-PE38 was purified by anion-exchange chromatography and size-exclusion chromatography. The half-maximal inhibitory concentration 50 of the immunotoxin, which was determined by the decrease in propidium iodine staining, was 10 ng/mL and 100 ng/mL for FRβ-transfected B300-19 cells (a murine pre-B cell line) and thioglycollate-elicited peritoneal macrophages from BALB/c or C57BL/6J mice, respectively. The recombinant immunotoxin contained <5 endotoxin units per milligram.

### Experimental Protocol of Immunotoxin Administration in a Mouse Model of Atherosclerosis

**Figure 1.**
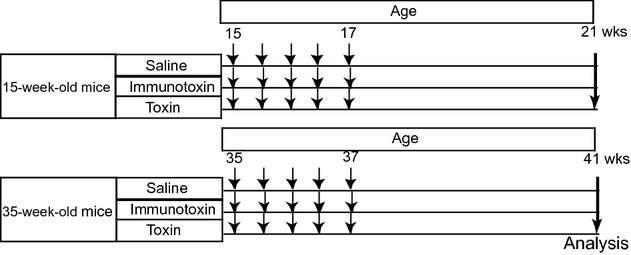
Time schedule of injections and tissue analyses. At 15 and 35 wk of age, mice were intravenously injected with 0.1 mg/kg of immunotoxin (immunotoxin group: 21 wk, n=8; 41 wk, n=5), 0.1 mg/kg of PE38 (toxin group: 21 wk, n=8; 41 wk, n=5), or 100 μL of saline (control group: 21 wk, n=8; 41 wk, n=5) every 3 days for a total of 5 times for each age group. At 21 and 41 wk of age, atherosclerotic lesions in the mice were analyzed. Small arrows indicate timing of injections.

We analyzed the effects of the recombinant immunotoxin on atherosclerosis in the male apoE-deficient mice (Figure 1). We divided the 15- and 35-week-old apoE-deficient mice into 3 groups: immunotoxin, toxin, and control (15 weeks: n=8 per group; 35 weeks: n=5 per group). During a period of 2 weeks, the immunotoxin group intravenously received 0.1 mg/kg of dsFv anti–FRβ-PE38 in 100 μL of saline, the toxin group received 0.1 mg/kg of PE38 in 100 μL of saline, and the control group received 100 μL of saline every 3 days, for a total of 5 times for each age group. The atherosclerotic lesions in the aortic roots were analyzed 1 week and 4 weeks later.

### Tissue Preparation and Lesion Assessment

The apoE-deficient mice were anesthetized with pentobarbital (80 μg/kg body weight, intraperitoneally), and blood samples were collected from the left ventricle. The number of leukocytes was counted by using a Coulter counter. Plasma cholesterol levels were measured with the use of an enzymatic kit (Kainos Laboratories, Tokyo, Japan). After intraventricular perfusion with 0.9% saline, the heart and ascending aorta were removed and quickly frozen in liquid nitrogen. Hearts were embedded in the Tissue-Tek OCT compound (Sakura Finetek Japan Co, Ltd, Tokyo, Japan), and 5-μm-thick cross sections of aortic roots were prepared, from the aortic valve leaflets through to the end of the atherosclerotic lesion. Every tenth section (50 μm apart) was stained with Oil Red O and hematoxylin. The atherosclerotic lesions were determined as the percentage of the lipid-stained area per vessel area, as described previously.^18–19^

### Immunohistochemistry

Immunohistochemical staining was performed as described previously.^20–21^ Acetone-fixed frozen sections (5 μm thick) were stained with a monoclonal rat anti–mouse CD68 antibody (AbD Serotec, Oxford, UK), monoclonal rat anti–mouse FRβ antibody, polyclonal rabbit anti–mouse tumor necrosis factor-α (TNF-α) antibody, α-smooth muscle actin (α-SMA), proliferating cell nuclear antigen (PCNA), rabbit anti–mouse CD31 (Santa Cruz Biotechnology, Santa Cruz, CA), rabbit anti–human CD3 (Dako, Kyoto, Japan), or isotype-matched irrelevant antibodies (AbD Serotec). After blocking with 3% skimmed milk, endogenous peroxidase activity was quenched with 0.3% H_2_O_2_ in methanol for 10 minutes, and slides were incubated overnight with a primary antibody at 4°C in a moisture chamber. Staining was performed with a MAX-PO secondary antibody (Nichirei Co, Ltd, Tokyo, Japan) and the NovaRed kit (Vector Laboratories, Burlingame, CA). Morphometric analysis was performed with a Zeiss Axioskop microscope (Carl Zeiss, Jena, Germany) and with Image J analysis software, and the percentage of plaque area exhibiting antibody staining was measured at 21 and 41 weeks of age.

Double immunofluorescence staining was performed on the frozen tissue sections. First, sections were reacted with the rat anti-FRβ mAb followed by the chicken anti–rat IgG–conjugated Alexa Fluor 594 (Molecular Probes, Eugene, OR). Sections then were incubated with one of the following: (1) Alexa Fluor 488–conjugated rat anti–mouse CD68 mAb (AbD Serotec, Raleigh, NC); (2) rabbit anti–mouse CD31 (Santa Cruz Biotechnology); (3) rabbit anti–human CD3 (Dako), followed by the goat anti–rabbit IgG–conjugated Alexa Fluor 488 (Molecular Probes); or (4) mouse anti–mouse SMA (Santa Cruz Biotechnology), followed by the goat anti–mouse IgG–conjugated Alexa Fluor 488 (Molecular Probes). Immunofluorescent double-stained sections were imaged with an LSM-700 confocal laser microscope (Carl Zeiss, Jena, Germany).

### Measurement of Apoptotic Cells

One week after the last injection, terminal deoxynucleotidyl-transferase–mediated nick-end labeling (TUNEL) was performed to detect apoptotic cells. Five-micrometer-thick cryostat sections were fixed in acetone, underwent staining with the DeadEnd Fluorometric TUNEL System (Promega, Madison, WI) according to the manufacturer's instructions, and were mounted with Vectashield mounting medium with DAPI (Vector Laboratories, Burlingame, CA). Control slides were treated with incubation buffer that did not contain the terminal deoxynucleotidyl transferase (TdT) enzyme. The TUNEL-positive cells were counted by using a BZ-8000 fluorescence microscope (Keyence, Osaka, Japan), and the percentage of cells undergoing apoptosis was calculated from the mean number of TUNEL-positive cells and the mean total number of cells from 3 randomly chosen fields from each section (18 weeks: n=8; 38 weeks: n=5).

### In Vitro Effect of FRβ Immunotoxin

Male apoE-deficient mice (n=3) at 12 weeks of age were intraperitoneally administered 3% of thioglycollate. Four days after injection, peritoneal cells were harvested with ice-cold phosphate-buffered saline, washed, and suspended in Iscove's modified Dulbecco's medium (Life Technologies, Carlsbad, CA) containing 10% fetal calf serum. After removal of nonadherent cells, macrophages were incubated with the same medium in the presence of either dsFv anti-FRβ or Ig V_H_-PE38 for 72 or 96 hours at 37°C in an atmosphere of 5% CO_2_. Propidium iodine (Sigma-Aldrich, Seelze, Germany) staining was performed as described previously.^12^ Briefly, cells were stained with propidium iodine in 0.1% sodium citrate and 0.1% Triton X-100, and apoptotic cells were determined by a decrease in propidium iodine staining as detected by flow cytometry. Induced apoptosis (%) was determined by subtracting the percentage of apoptotic cells caused by IgV_H_-PE38 from that of dsFv anti-FRβ in each sample. To analyze the FRβ expression on thioglycollate-elicited peritoneal cells, adherent cells were stained with allophycocyanic-conjugated anti-FRβ mAb, fluorescein isothiocyanate–F4/80 mAb (Biolegend, San Diego, CA), or allophycocyanic- or fluorescein isothiocyanate–conjugated isotype control mAb (Biolegend).

### Quantitative Real-Time PCR

Quantitative real-time PCR (RT-PCR) with the TaqMan Probe method was performed to determine the differential expression of FRβ and TNF-α mRNAs relative to the expression of glyceraldehydephosphatedehydrogenase (GAPDH) mRNA. Total RNA was extracted from the microdissected aorta by using the RiboPure Kit (Ambion, Austin, TX), followed by reverse transcription of the isolated RNA with the use of a high-capacity cDNA Reverse Transcription Kit (Applied Biosystems, Foster City, CA). The first-strand cDNA was used as a template, and RT-PCR was performed with the use of the ABI PRISM 7300 Sequence Detection System (Applied Biosystems) with TaqMan Universal PCR Master Mix (Applied Biosystems). Primers and probes for FRβ, TNF-α, and GAPDH were purchased from Applied Biosystems. Primer sequences for FRβ were 5′-AAGGCTGACTCCCGTCTGTA-3′ (forward) and 5′-GCTCTTTACGCCAACTCTGG-3′ (reverse), and the probe sequence was 5′-TAAGAGTCACTTCATCCAAG-3′.

### Statistical Analysis

Data are presented as the standard error of the mean (SEM). For comparison of multiple independent groups, Kruskal-Wallis test followed by Holm's pairwise comparison was used. For the assessment of treatment effect and age relationships, data were analyzed with a 2-way factorial analysis of variance. A paired Student *t* test was used to compare the in vitro effect of immunotoxin and toxin. A probability value of *P*<0.05 was considered statistically significant.

## Results

### FRβ Expression in Atherosclerotic Lesions

**Figure 2.**
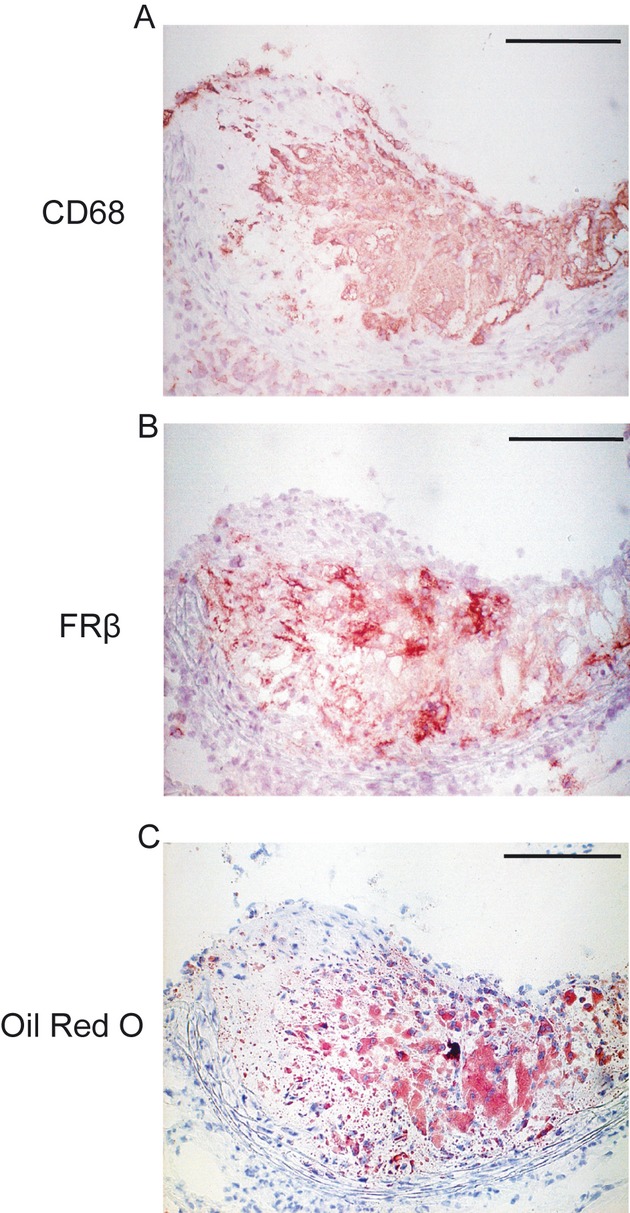
Immunohistochemistry of FRβ and CD68 in 21-week-old mice. Representative photographs of CD68 (A) and FRβ (B) immunostaining and Oil Red O (C) staining of serial sections from the aortic sinus in the apoE-deficient mice (scale bar=100 μm). FRβ-expressing cells were detected mainly in the intimal elastic lamina side of the intima, where macrophages tend to be larger. FRβ, folate receptor-β.

**Figure 3.**
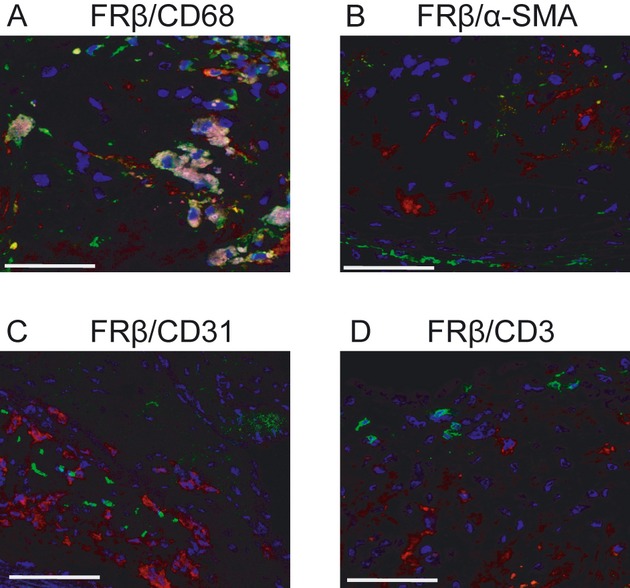
Representative double fluorescence immunohistochemistry of aortic cross sections at 21 wk of age. Representative photographs of FRβ-expressing cells in atherosclerotic lesions. Cryosectioned aortic sinus lesions were stained with (A) anti-CD68 (green) and anti-FRβ followed by Alexa Fluor 594 (red), (B) anti-αSMA (green) and FRβ (red), (C) anti-CD31 (green) and FRβ (red), or (D) anti-CD3 (green) and FRβ (red). FRβ was colocalized with CD68 but not with CD31, α-SMA, or CD3 (scale bar=50 μm). FRβ, folate receptor-β; α-SMA, α-smooth muscle actin.

We first evaluated the presence and localization of FRβ-expressing cells in the aortic sinus lesion of the apoE-deficient mice at 21 weeks of age. We compared the distribution of Oil Red O staining to that of FRβ-expressing cells in the serial slides (Figure 2A through 2C). Of foam cells, 91.2±2.4% expressed FRβ, and of non–lipid-loaded macrophages, 4.6±1.2% expressed FRβ. Many of the FRβ-expressing cells were observed in the intimal elastic lamina side of the intima, where macrophages tended to be larger than those of the luminal side. At 21 weeks of age, double fluorescence immunohistochemistry demonstrated colocalization of FRβ with CD68 (Figure 3A), but FRβ was not colocalized with α-SMA, CD31, or CD3 (Figure 3B through 3D). The percentages of FRβ-expressing cells among the macrophage population were 57.2±2.9% at 21 weeks and 69.7±3.0% at 35 weeks of age.

### FRβ Immunotoxin Administration Attenuates Progression of Atherosclerosis

**Figure 4.**
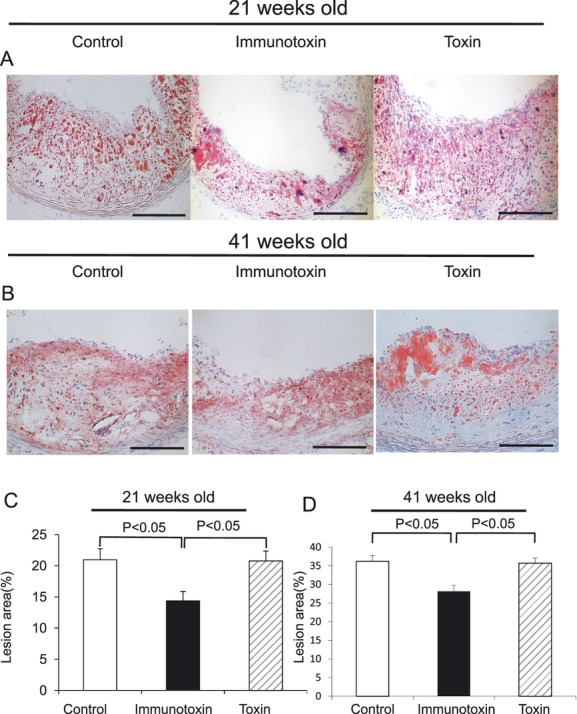
Representative Oil Red O staining of aortic cross sections of control, immunotoxin, and toxin groups at the 21- and 41-week time points. The atherosclerotic lesions in the immunotoxin group were smaller than those in the control and toxin groups at 21 wk (n=8 per group) (A) and 41 wk of age (n=5 per group) (B) (scale bar=100 μm). Quantification of the atherosclerotic volume in the aortic roots demonstrated that the administration of FRβ immunotoxin reduced the atherosclerotic lesions of 21-week-old (C) and 41-week-old (D) apoE-deficient mice. FRβ, folate receptor-β; apoE, apolipoproteinE.

We used younger and older mice of 15 and 35 weeks of age in these studies. Each age group was divided into immunotoxin, toxin, and control groups and received the dsFv anti–FRβ-PE38 immunotoxin, PE38, or saline, respectively, every 3 days for a total of 5 times. When the mice were euthanized at 21 and 41 weeks of age, the immunotoxin group showed a 31% and 22% reduction in atherosclerotic lesions, respectively (Figure 4A and 4B), compared with the control and toxin groups (21 weeks: control=20.9±1.7%, immunotoxin=14.4±1.4%, toxin=20.8±1.5%, *P*=0.03, n=8; 41 weeks:control=36.2±1.5%, immunotoxin=28.1±5.6%, toxin=35.7±1.3%, *P*=0.02, n=5) (Figure 4C and 4D). Treatment with PE38 had no effect on the size of atherosclerotic lesions, and there was no interaction between age and the effect of the treatments. No significant differences were observed among the 3 groups in body weights, serum levels of total cholesterol, or numbers of circulating neutrophils, eosinophils, monocytes, and lymphocytes in peripheral blood. We also used 2-factor analysis of variance for several measurements that might affect the differences by treatment in the 2 age groups, such as body weights, total cholesterol levels, the number of monocytes, and the number of eosinophils. Of these data, there were significant differences in the number of eosinophils and monocytes between the 2 age groups. However, immunotoxin or toxin had no effect on the numbers of eosinophils or monocytes, and there was no interaction between age and the drug effect. (Table 1).

**Table 1.** Body Weight, Total Cholesterol Levels, and Peripheral White Blood Cells

	Control	Immunotoxin	Toxin
21 wk			
Body weight, g	26.9±0.7	25.7±0.9	28.0±0.6
Total cholesterol, mg/dL	530.5±42	495.0±40	488.7±55
Neutrophils, /μL	1608±320	1444±302	1640±205
Eosinophils, /μL	46±8	43±10	38±10
Monocytes, /μL	119±27	127±33	102±35
Lymphocytes, /μL	2398±273	2296±381	2137±342
41 wk			
Body weight, g	28.1±0.7	27.2±1.1	27.0±1.1
Total cholesterol, mg/dL	611.6±66	577.2±57	569.2±57
Neutrophils, /μL	1099±267	1120±207	1201±305
Eosinophils, /μL	55±9	63±10	69±11
Monocytes, /μL	62±18	54±8	52±7
Lymphocytes, /μL	2088±482	1978±52	1811±301

There were no significant differences in body weights (21 wk: n=8; 41 wk: n=5), total cholesterol levels, or peripheral white blood cell numbers among the control, immunotoxin, and toxin groups of apoE-deficient mice at the 21- and 41-week time points. apoE, apolipoproteinE.

### Immunohistochemical Analysis of Atherosclerotic Lesions After FRβ Immunotoxin Administration

**Figure 5.**
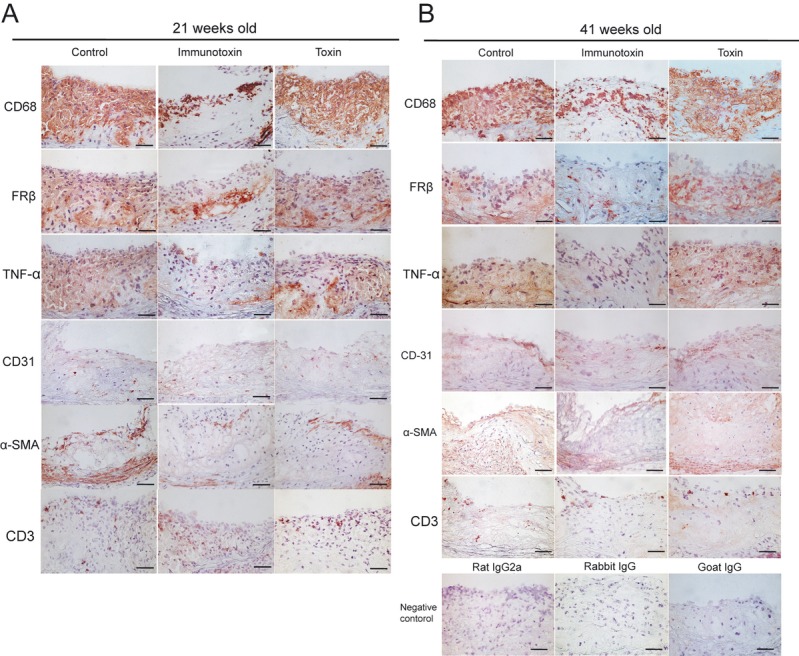
Immunohistochemical evaluation of atherosclerotic lesions. Cryosectioned aortic sinus lesions were stained with antibodies directed against CD68, FRβ, TNF-α, CD31, α-SMA, and CD3. The percentages of CD68-, FRβ-, and TNF-α–stained area were significantly reduced in the immunotoxin group compared with the toxin or control group at both 21 and 41 wk of age (21 wk: n=8; 41 wk: n=5 per group). FRβ, folate receptor-β; TNF-α, tumor necrosis factor-α; α-SMA, α-smooth muscle actin.

Administration of FRβ immunotoxin significantly reduced the percentage of CD68-, FRβ-, and TNF-α–stained area compared with the toxin or control group at both 21 and 41 weeks of age (Figure 5A and 5B). There was a slight decrease in the percentage of FRβ-expressing cells in the toxin group compared to the control group (*P*=0.04) at 41 weeks of age only. This observation could indicate the possibility of a nonspecific effect of the toxin on phagocytic cells.^22^ Although α-SMA–expressing cells tended to decrease in the immunotoxin groups at 21 and 41 weeks of age, these differences did not reach statistical significance, and there was no significant difference in CD3-expressing cells (Table 2).

**Table 2.** Immunohistochemical Analysis of Atherosclerotic Lesions

	Control	Immunotoxin	Toxin
21 wk			
CD68	64.7±4.3	25.3±4.8*†	69.7±3.7
FRβ	51.7±4.6	22.8±4.2*†	54.9±6.0
TNF-α	42.7±8.6	11.5±2.3*†	35.7±5.2
CD3	6.0±0.8	8.7±1.9	7.4±1.3
α-SMA	10.7±1.7	8.0±0.9	10.8±0.7
PCNA	20.2±1.7	11.5±1.1*†	16.7±0.6
TUNEL	16.5±1.4	28.1±1.7*†	14.0±1.9
41 wk			
CD68	52.2±5.6	20.8±4.3*†	57.0±8.9
FRβ	68.5±4.5	38.0±4.1*†	56.4±2.3*
TNF-α	68.8±4.3	47.9±2.4*†	65.5±3.3
CD3	5.6±1.0	7.7±1.9	6.3±1.1
α-SMA	9.1±0.7	6.4±1.2	9.8±1.2
PCNA	14.2±0.5	8.7±0.4*†	13.9±1.3
TUNEL	12.7±1.2	28.9±2.9*†	15.8±2.1

Administration of FRβ immunotoxin significantly reduced the percentage of CD68-, FRβ-, and TNF-α–stained area compared with the toxin or control group in both 21- and 41-week-old mice (21 wk: n=8; 41 wk: n=5). The percentage of apoptotic cells in the immunotoxin group was significantly higher than that in control and toxin groups. The percentage of cells expressing PCNA was significantly decreased compared with control and toxin groups in both age groups. TUNEL and PCNA staining was performed 1 wk after the last injection (18 wk: n=8; 38 wk: n=5). FRβ, folate receptor-β; TNF-α, tumor necrosis factor-α; α-SMA, α-smooth muscle actin; PCNA, proliferating cell nuclear antigen; TUNEL, terminal deoxynucleotidyltransferase–mediated nick-end labeling.

* *P*<0.05 vs control group.

† *P*<0.05 vs toxin group.

### Effects of FRβ Immunotoxin on Cell Proliferation and Apoptosis in Atherosclerotic Lesions

**Figure 6.**
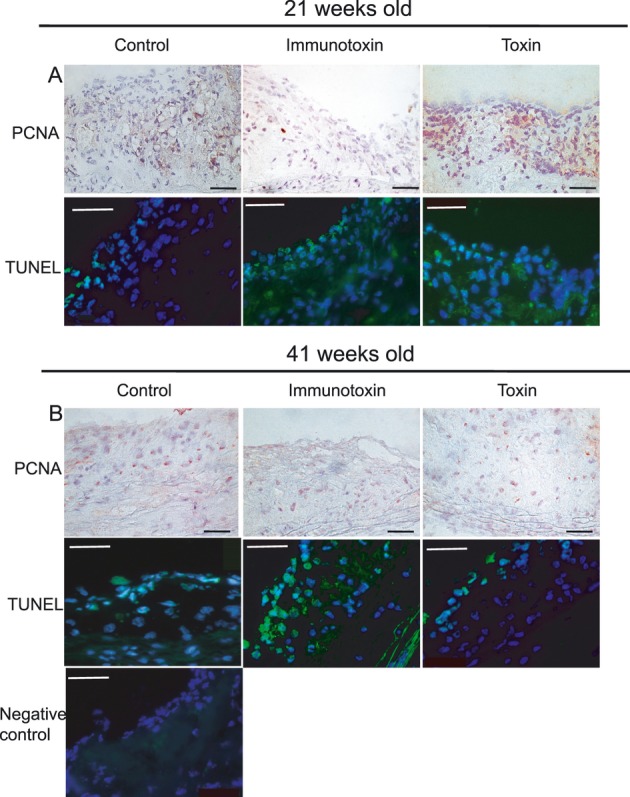
Effects of FRβ immunotoxin on cell proliferation and apoptosis in atherosclerotic lesions. Representative photographs of cells stained with anti-PCNA antibody and TUNEL staining in the aortic roots of apoE-deficient mice 1 wk after the last injection at 18 wk (A) and 38 wk (B) of age (scale bar=50 μm). The percentage of cells expressing PCNA was significantly decreased compared with control and toxin groups in both age groups. The percentage of apoptotic cells in the immunotoxin group was significantly higher than that in the control and toxin groups (18 wk: n=8; 38 wk: n=5). FRβ, folate receptor-β; apoE, apolipoproteinE; PCNA, proliferating cell nuclear antigen; TUNEL, terminal deoxynucleotidyltransferase–mediated nick-end labeling.

Because the effect of immunotoxin on apoptosis wears off within a few days and apoptosis cells induced by immunotoxin were not detected 4 weeks after the last injection (probably because of the removal of apoptotic cells), PCNA and TUNEL staining was performed 1 week after the last injection to assess the level of cell proliferation and apoptosis (Figure 6). As shown in Table 2, the percentage of apoptotic cells in the immunotoxin group was significantly higher than that in the control and toxin groups. Also, the percentage of cells expressing PCNA was significantly decreased compared with the control and toxin groups in both age groups.

### In Vitro Effect of FRβ Immunotoxin

**Figure 7.**
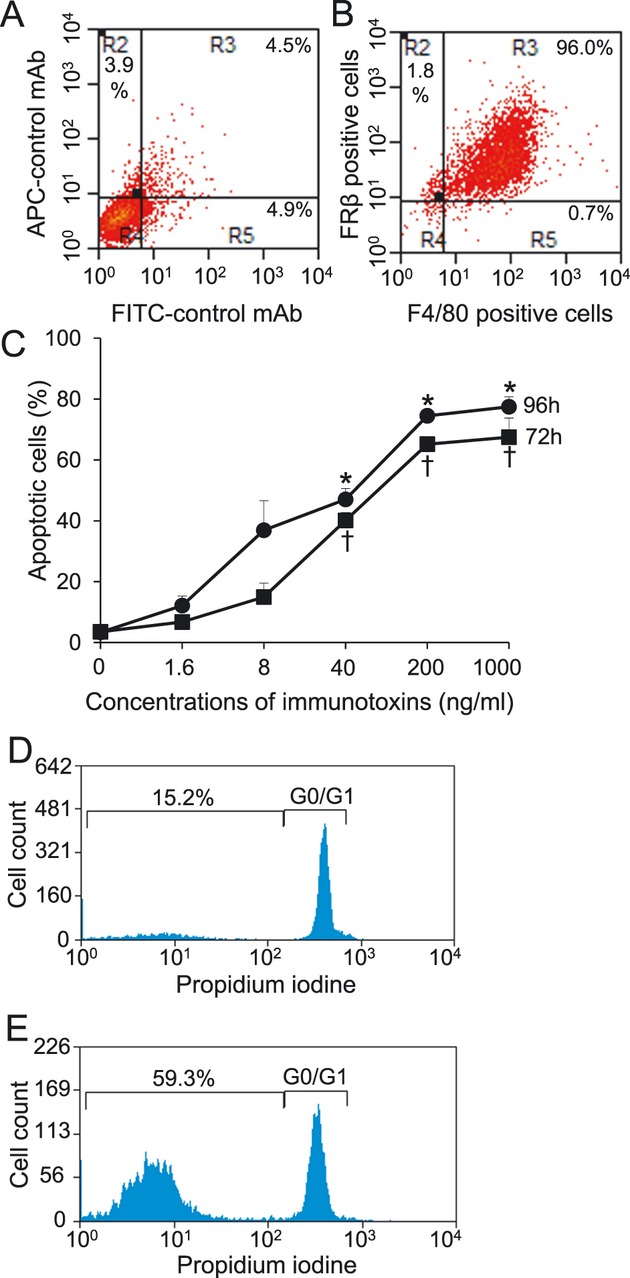
In vitro effect of FRβ immunotoxin. Thioglycollate-elicited peritoneal macrophages were stained with allophycocyanin-conjugated isotype control mAb and fluorescein isothiocyanate–conjugated isotype control mAb (A) or allophycocyanic–anti-FRβ mAb and fluorescein isothiocyanate–F4/80 mAb (B). The percentage of peritoneal macrophages expressing FRβ was 95.4±1.3%. Flow cytometry analysis of apoptosis in thioglycollate-elicited peritoneal macrophages by propidium iodine staining. Cells were cultured with the designated concentration of dsFv anti–FRβ-PE38 or V_H_-PE38 for 72 or 96 hours. The immunotoxin induced apoptosis in peritoneal macrophages with IC_50_ of 40 ng/mL at 72 hours (square) and 96 hours (circle) (C). Representative images of thioglycollate-elicited peritoneal macrophages with V_H_-PE38 (D) or dsFv anti–FRβ-PE38 (E) for 72 hours at the concentrations of 40 ng/mL, respectively (n=3 per group). FRβ, folate receptor-β. *P<0.05 vs toxin group at 96 hours. †*P*<0.05 vs toxin group at 72 hours.

We stained thioglycollate-elicited peritoneal cells by using anti-FRβ mAb and anti-F4/80 mAb, and the percentage of peritoneal macrophages expressing FRβ was 95.4±1.3% (Figure 7A). To confirm the in vitro effect of FRβ immunotoxin on apoptosis, thioglycollate-elicited peritoneal macrophages cultured in the presence of either dsFv anti–FRβ-PE38 or V_H_-PE38 were stained with propidium iodine, and the presence of apoptotic cells was measured by flow cytometry. The immunotoxin induced apoptosis in peritoneal macrophages with an half maximal inhibitory concentration (IC_50_) of 40 ng/mL at 72 and 96 hours (Figure 7B), and the percentages of apoptotic cells at the concentration of 1000 ng/mL were 67.4±6.3% for 72 hours and 77.4±3.3% for 96 hours (Figure 7C through 7E).

### RT-PCR Analysis of mRNA Expression of FRβ and TNF-α in Atherosclerosis After FRβ Immunotoxin Administration

**Figure 8.**
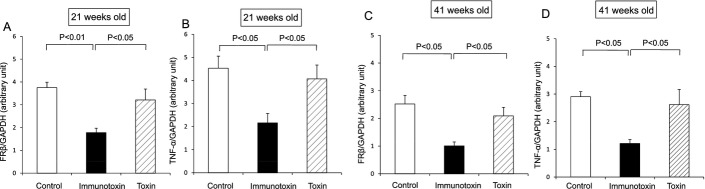
Effects of FRβ immunotoxin on FRβ and TNF-α mRNA expression. The expression of FRβ and TNF-α mRNA was quantified by RT-PCR. Administration of FRβ immunotoxin significantly decreased the expression of FRβ mRNA compared to the control and toxin groups at 21 wk (A) and 41 wk of age (C). Similarly, the expression of TNF-α mRNA in the immunotoxin group also decreased significantly at the 21-week (B) and 41-week (D) time points compared to the control and toxin groups (21 wk: n=8; 41 wk: n=5). FRβ, folate receptor-β; TNF-α, tumor necrosis factor-α; RT-PCR, quantitative real-time polymerase chain reaction.

We analyzed FRβ and TNF-α mRNA expression in the aorta by quantitative RT-PCR. At 21 and 41 weeks of age, FRβ mRNA expression in the immunotoxin group was decreased significantly compared with the toxin or control groups (21 weeks: control=3.8±0.2, immunotoxin=1.8±0.2, toxin=3.2±0.5 arbitrary units, *P*=0.001, n=8; 41 weeks: control=2.5±0.3, immunotoxin=1.0±0.1, toxin=2.1±0.3 arbitrary units, *P*=0.02, n=5) (Figure 8). At 21 and 41 weeks of age, immunotoxin administration also reduced TNF-α mRNA expression compared with the toxin and control groups (21 weeks: control=4.5±0.5, immunotoxin=2.2±0.4, toxin=4.1±0.6 arbitrary units*, P*=0.01, n=8; 41 weeks: control=2.9±0.2, immunotoxin=1.2±0.1, toxin=2.6±0.6 arbitrary units, *P*=0.005, n=5) (Figure 8).

## Discussion

This study is the first demonstration that FRβ-expressing macrophages are present in the atherosclerotic lesions and that intravenous administration of FRβ immunotoxin markedly reduced the progression of atherosclerotic lesions in both the early and advanced stages of atherosclerosis. It has been reported previously that FRβ is expressed on activated macrophages at the site of inflammation.^11^ Previous work that used a polyclonal antibody to FRβ suggested that FRβ was a neutrophilic lineage marker.^23^ However, we used a mAb specific to FRβ and reported that FRβ was not expressed by peripheral blood neutrophils, even after in vitro stimulation with phorbol myristate acetate.^12^ In the present study, we demonstrated significant coexpression of FRβ with macrophages, but not with endothelial cells, vascular smooth muscle cells, or T cells. Moreover, histological analysis revealed that FRβ immunotoxin treatment resulted in a significant reduction of macrophages expressing CD68, FRβ, and TNF-α. In addition, the administration of FRβ immunotoxin also increased the percentage of TUNEL-positive apoptotic cells and reduced the percentage of PCNA-positive proliferating cells. RT-PCR performed on aorta demonstrated reduced mRNA expression of FRβ and TNF-α in mice treated with immunotoxin, which is in accordance with the histological data.

Macrophages play an important role in atherosclerosis by promoting inflammatory responses. Although targeting macrophages in atherosclerotic lesions seems to be a promising strategy for preventing the progression of atherosclerosis, the antiatherogenic effect of killing macrophages within the plaques remains controversial.^6^, ^24–27^ The difference in the phagocytic clearance of dead macrophages might lead to conflicting results according to the lesion stage. In the early stage of atherosclerosis, induction of macrophage apoptosis is beneficial because the phagocytic clearance of apoptotic macrophages is efficient during this stage.^24^ In contrast, in the advanced stage of atherosclerosis, increasing macrophage apoptosis is detrimental because of the defective phagocytic clearance of accumulated dead macrophages.^26–27^ Induction of macrophage apoptosis in the advanced stage could lead to the progression of atherosclerotic lesions, but previous studies targeted pan-macrophage. We targeted a selected subset of macrophages that engage inflammation and demonstrated that the dsFv anti-FRβ immunotoxin caused apoptosis in the thioglycollate-elicited peritoneal macrophages. In addition, administration of dsFv anti-FRβ immunotoxin increased apoptosis and suppressed the progression of atherosclerotic lesions in both the early and advanced stages of atherosclerosis. The majority of apoptotic cells in atherosclerotic lesions are macrophages,^4^ and cellular proliferation within atheroma involves predominantly vascular smooth muscle cells and macrophages.^28–29^ By induction of apoptosis in FRβ-expressing macrophages, growth factors and chemokines, which stimulate the proliferation of macrophages and smooth muscle cells, might decrease. Although there was not a significant difference in the number of smooth muscle cells between the control, immunotoxin, and toxin groups, the number of smooth muscle cells tended to decrease in the immunotoxin group. Taken together, elimination of a selected subset of macrophages that engage actively in inflammation might leave the clearance of apoptotic cells undisturbed, lead to the suppression of cell proliferation, and thus, exert favorable suppressive effects on the progression of atherosclerotic lesions in both the early and late stages of atherosclerosis.

In the immunohistochemical study, FRβ-expressing macrophages were observed mainly on the medial side of the intima, where macrophages tended to be larger than those on the luminal side. Although most of the FRβ-expressing cells were CD68 positive, some of them were CD68 negative. These FRβ^+^/CD68^−^ cells were noted to be anucleate and exhibited patchy deposits of Oil Red O staining, and they thus could represent the cellular remnants of dead macrophages. CD68 is expressed strongly in cytoplasmic granules of cells of the monocyte/macrophage lineage, whereas FRβ is expressed on the cell membrane. The difference in staining pattern between CD68 and FRβ should be able to explain the presence of CD68^−^/FRβ^+^ cells observed at anucleate area.

FRβ-expressing macrophages were reported to have produced TNF-α and reactive oxygen species^14^ and to act as M1 macrophages in rheumatoid arthritis.^30^ However, it remains controversial whether FRβ expression reflects M1 function because FRβ macrophages are considered to be M2 macrophages in glioma^16^ and melanoma.^31^ The patterns of gene expression of M1/M2 macrophages vary in response to various environmental stimuli, and FRβ in atherosclerotic lesions is heterogeneous and does not precisely fit either M1 or M2 in vivo. Although phenotypic variation among activated macrophages has not yet been completely validated, FRβ-expressing macrophages expressing TNF-α are often noted to be in lipid-laden macrophages, which eventually develop into foam cells. Targeting these FRβ-expressing macrophages might be an effective strategy to suppress inflammation in atherosclerotic lesions.

Several immunotoxins consisting of mAbs conjugated to toxins have been used for clinical trials, such as in acute lymphoblastic leukemia,^32^ hairy cell leukemia,^33^ and mesothelioma.^34^ The truncated *Pseudomonas aeruginosa* exotoxin known as PE38 lacks its binding domain.^35^ Our recombinant dsFv anti–FRβ-PE38 immunotoxin consisted of the truncated PE38 and the heavy- and light-chain portions of the anti-FRβ antibody. When FRβ-expressing macrophages internalize the immunotoxin, the PE fragments travel through the endosomes into the trans-Golgi network. The PE fragments then are secreted into the cytosol, where they inhibit protein synthesis by elongation factor-2, leading to apoptosis.^36–37^ Because FRβ is expressed exclusively on activated macrophages in atherosclerotic lesions, FRβ immunotoxin could be capable of mediating macrophage apoptosis in patients with ischemic heart diseases and peripheral arterial diseases thorough its specificity and cytotoxicity. Several problems such as nonspecific toxicity^32,38^ and immunogenicity^39^ should be solved before the clinical use of the immunotoxins can be adopted. Although elevation of hepatic transaminases was observed as an adverse effect of nonspecific toxicity, this proved to be reversible and had an acceptable safety profile.^32^ In addition, the humanized immunotoxins, with their cytotoxic moiety based on proteins or RNase of human origin,^40–42^ might overcome problems with immunogenicity.

Several attempts have been made to inhibit the development of atherosclerotic lesions by administering antibodies against mediators such as placental growth factor, CD40L, macrophage colony–stimulating factor, and monocyte chemoattractant proteins 1 and 5.^43–46^ These interventions have induced regression of atherosclerotic lesions by blocking certain epitopes or chemokines on macrophages and vascular smooth muscle cells, resulting in reduced macrophage recruitment and the downregulation of proinflammatory pathways. In contrast to those studies, we used FRβ immunotoxin to induce apoptosis of activated macrophages rather than targeting individual chemokines. The elimination of activated FRβ-expressing macrophages could prevent activated macrophages from releasing inflammatory cytokines and recruiting circulating macrophages and thus could break the vicious circle operating in the atherogenic environment.

Previous reports have indicated that folate conjugates detect sites of inflammation in adjuvant-induced arthritis and atherosclerosis, which involve activated macrophages.^15,47–48^ Although the uptake of folate conjugates was mediated by the FR, it is possible that other FR isoforms were involved. In the present study, we targeted the β isoform of FR that is only expressed by activated macrophages. We believe that the cell specificity of this method might be of particular value in delivering imaging or therapeutic agents to atherosclerotic lesions. We found that FRβ was expressed by macrophages in the atherosclerotic lesions of human carotid and renal arteries (data not shown). The FRβ antibody could be used as an imaging agent to distinguish vulnerable plaques in human atherosclerosis that are prone to rupture, resulting in myocardial infarction.

In conclusion, we have demonstrated the presence and distribution of FRβ-expressing macrophages in atherosclerotic lesions. A recombinant FRβ immunotoxin reduced FRβ and TNF-α mRNA expression and the number of FRβ- and TNF-α–expressing macrophages and thus suppressed the progression of atherosclerotic lesions. Targeting activated macrophages with the FRβ immunotoxin could be beneficial in the suppression of both the early and advanced stages of atherosclerosis. Although further clinical research would be required to confirm the effect of killing activated macrophages in human atherosclerotic lesions, our findings provide a new strategy for targeting activated macrophages, with clinical implications for the treatment of atherosclerosis.
